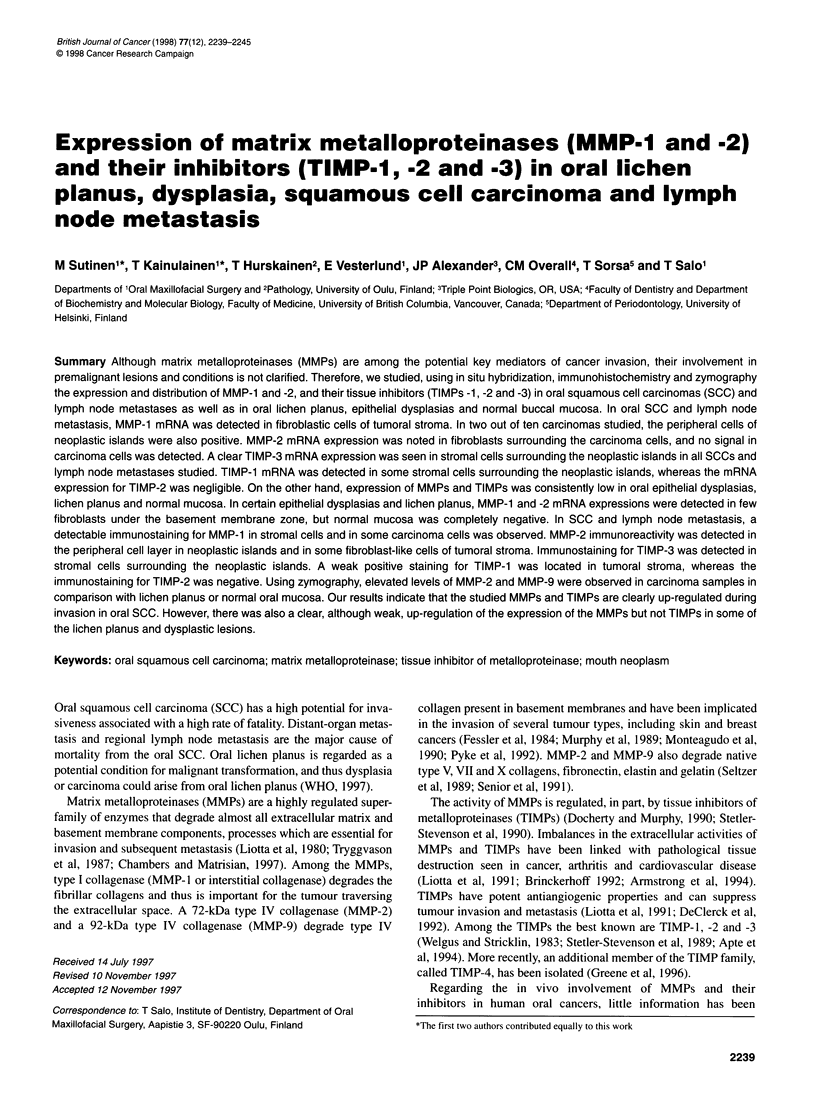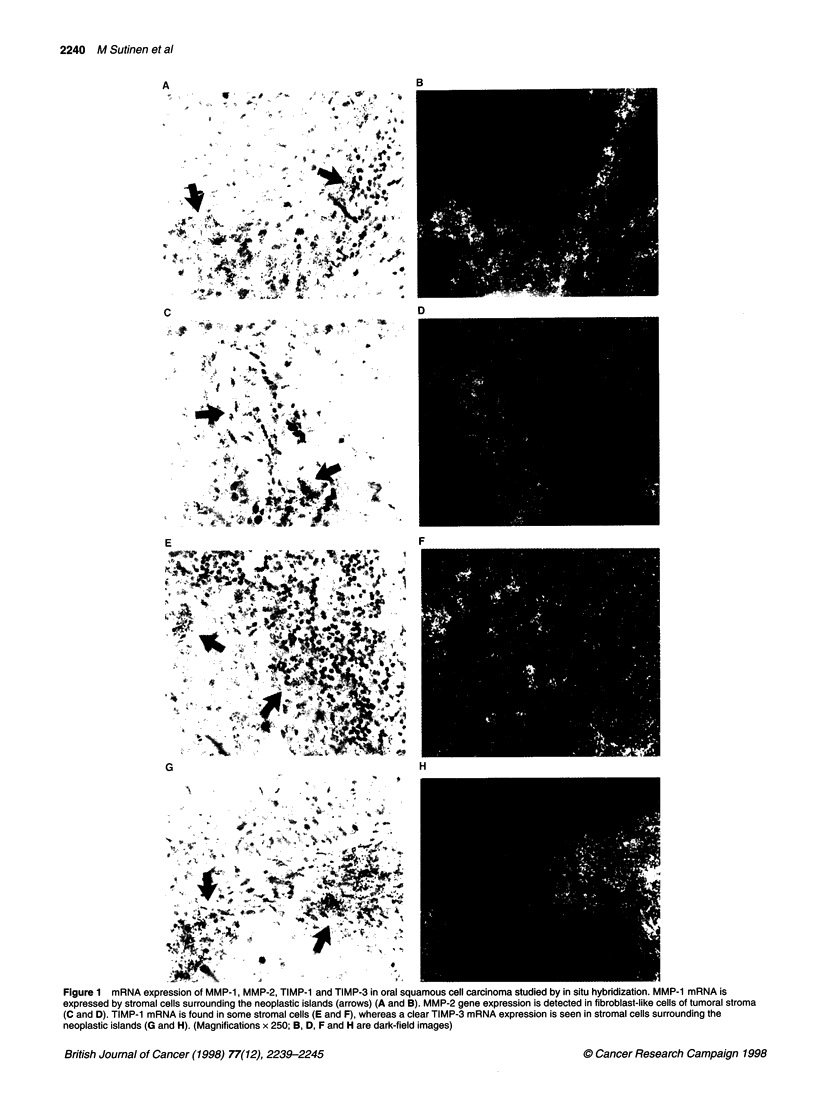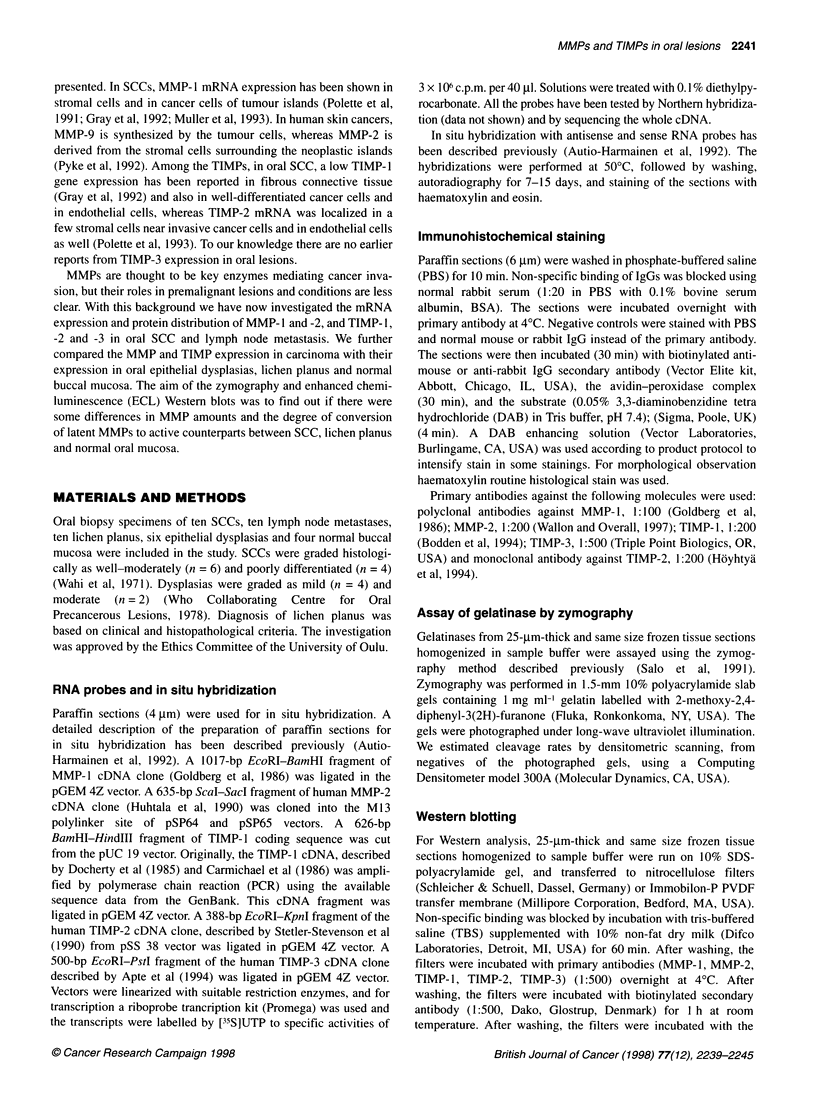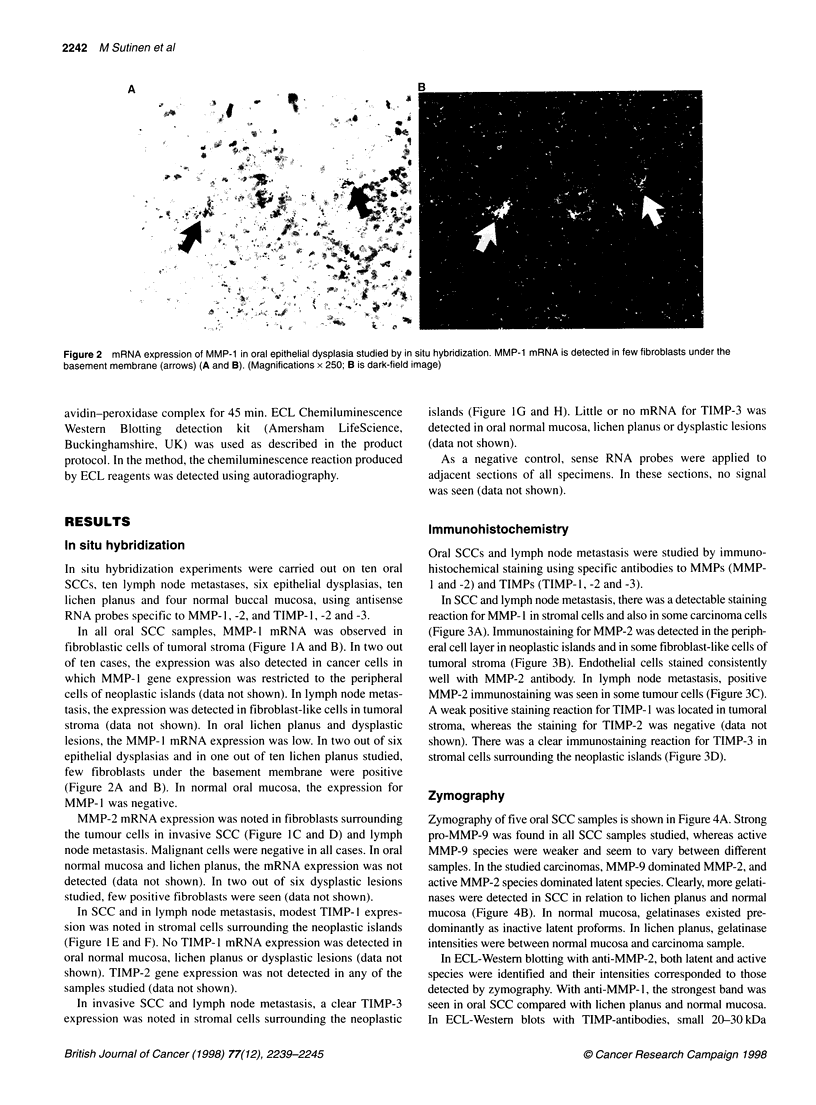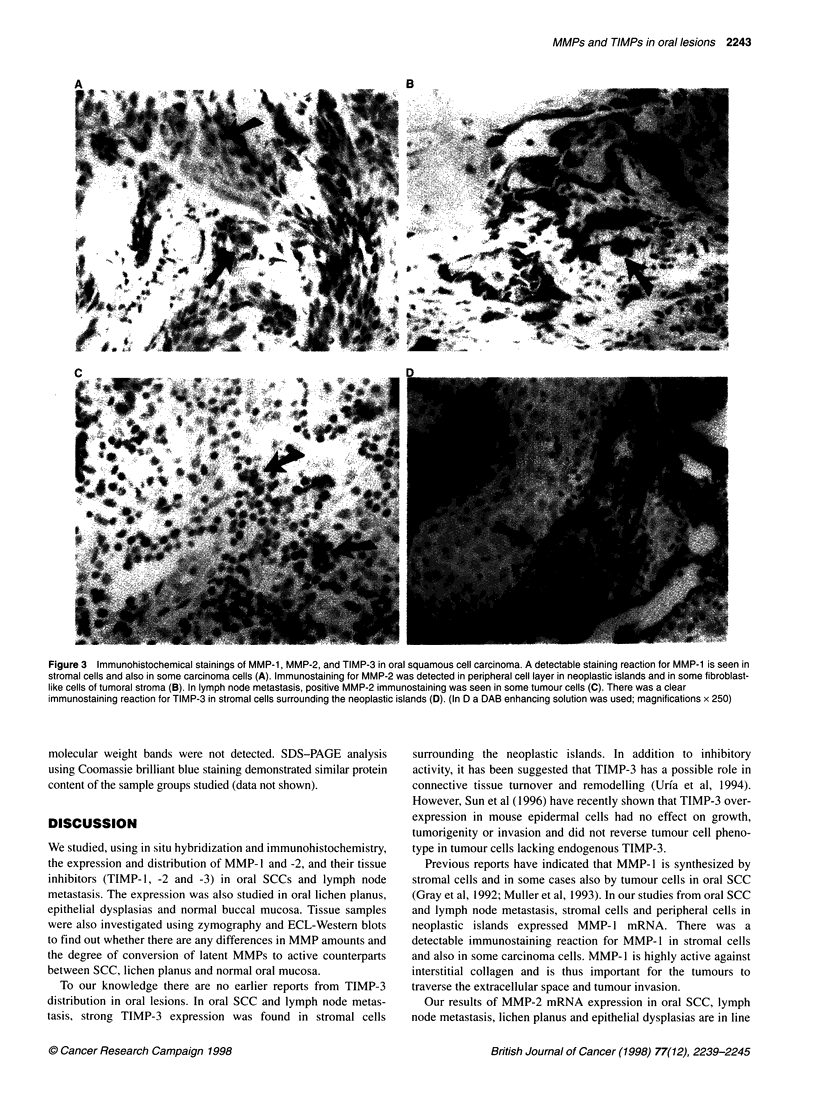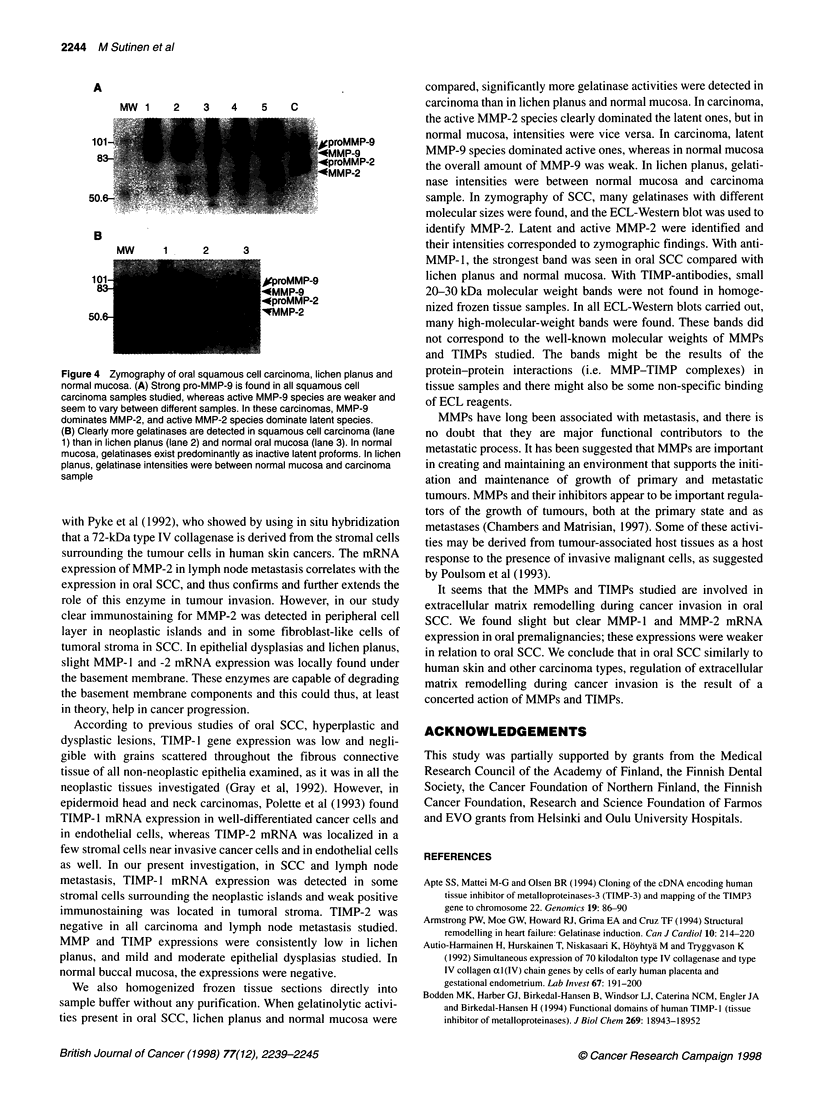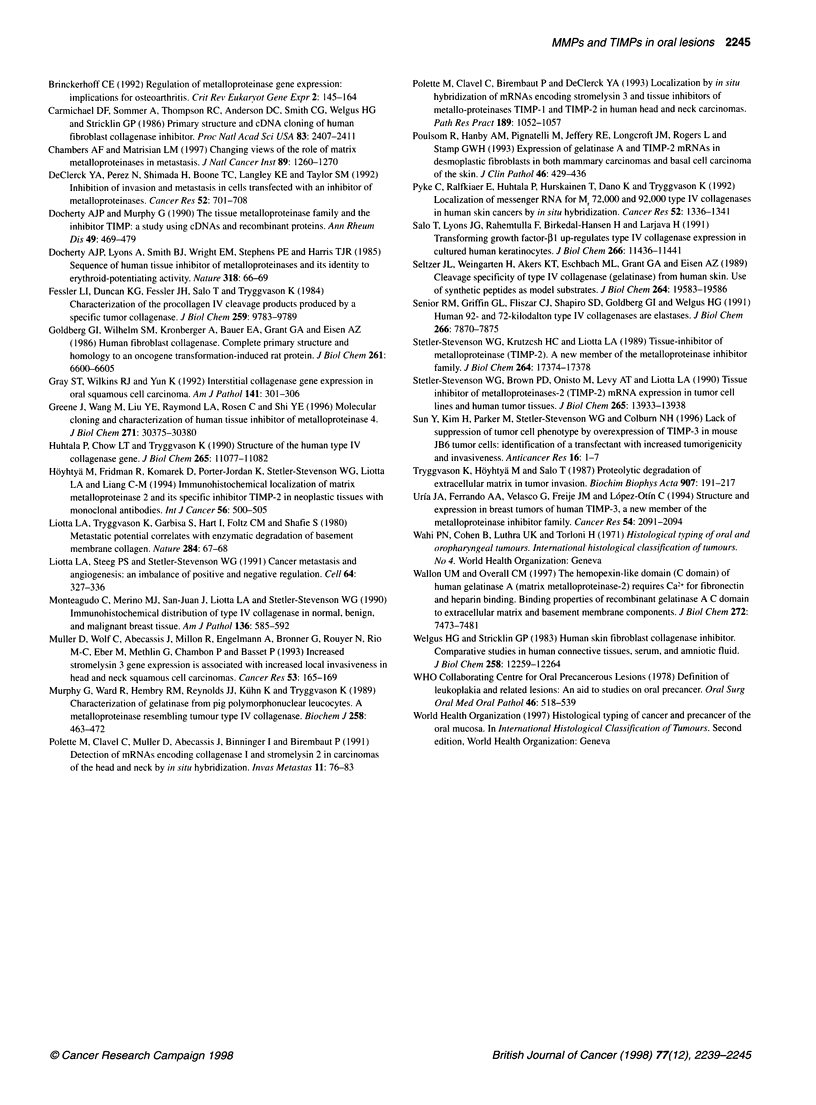# Expression of matrix metalloproteinases (MMP-1 and -2) and their inhibitors (TIMP-1, -2 and -3) in oral lichen planus, dysplasia, squamous cell carcinoma and lymph node metastasis.

**DOI:** 10.1038/bjc.1998.372

**Published:** 1998-06

**Authors:** M. Sutinen, T. Kainulainen, T. Hurskainen, E. Vesterlund, J. P. Alexander, C. M. Overall, T. Sorsa, T. Salo

**Affiliations:** Department of Oral Maxillofacial Surgery, University of Oulu, Finland.

## Abstract

**Images:**


British Journal of Cancer (1998) 77(12), 2239–2245
© 1998 Cancer Research Campaign

### Expression of matrix metalloproteinases (MMP-1 and -2) and their inhibitors (TIMP-1, -2 and -3) in oral lichen planus, dysplasia, squamous cell carcinoma and lymph node metastasis

**M Sutinen[1]*, T Kainulainen[1]*, T Hurskainen[2], E Vesterlund[1], JP Alexander[3], CM Overall[4], T Sorsa[5] and T Salo[1]**

Departments of [1]Oral Maxillofacial Surgery and [2]Pathology, University of Oulu, Finland; [3]Triple Point Biologics, OR, USA; [4]Faculty of Dentistry and Department of Biochemistry and Molecular Biology, Faculty of Medicine, University of British Columbia, Vancouver, Canada; [5]Department of Periodontology, University of Helsinki, Finland

**Summary** Although matrix metalloproteinases (MMPs) are among the potential key mediators of cancer invasion, their involvement in premalignant lesions and conditions is not clarified. Therefore, we studied, using in situ hybridization, immunohistochemistry and zymography the expression and distribution of MMP-1 and -2, and their tissue inhibitors (TIMPs -1, -2 and -3) in oral squamous cell carcinomas (SCC) and lymph node metastases as well as in oral lichen planus, epithelial dysplasias and normal buccal mucosa. In oral SCC and lymph node metastasis, MMP-1 mRNA was detected in fibroblastic cells of tumoral stroma. In two out of ten carcinomas studied, the peripheral cells of neoplastic islands were also positive. MMP-2 mRNA expression was noted in fibroblasts surrounding the carcinoma cells, and no signal in carcinoma cells was detected. A clear TIMP-3 mRNA expression was seen in stromal cells surrounding the neoplastic islands in all SCCs and lymph node metastases studied. TIMP-1 mRNA was detected in some stromal cells surrounding the neoplastic islands, whereas the mRNA expression for TIMP-2 was negligible. On the other hand, expression of MMPs and TIMPs was consistently low in oral epithelial dysplasias, lichen planus and normal mucosa. In certain epithelial dysplasias and lichen planus, MMP-1 and -2 mRNA expressions were detected in few fibroblasts under the basement membrane zone, but normal mucosa was completely negative. In SCC and lymph node metastasis, a detectable immunostaining for MMP-1 in stromal cells and in some carcinoma cells was observed. MMP-2 immunoreactivity was detected in the peripheral cell layer in neoplastic islands and in some fibroblast-like cells of tumoral stroma. Immunostaining for TIMP-3 was detected in stromal cells surrounding the neoplastic islands. A weak positive staining for TIMP-1 was located in tumoral stroma, whereas the immunostaining for TIMP-2 was negative. Using zymography, elevated levels of MMP-2 and MMP-9 were observed in carcinoma samples in comparison with lichen planus or normal oral mucosa. Our results indicate that the studied MMPs and TIMPs are clearly up-regulated during invasion in oral SCC. However, there was also a clear, although weak, up-regulation of the expression of the MMPs but not TIMPs in some of the lichen planus and dysplastic lesions.

**Keywords:** oral squamous cell carcinoma; matrix metalloproteinase; tissue inhibitor of metalloproteinase; mouth neoplasm

Oral squamous cell carcinoma (SCC) has a high potential for invasiveness associated with a high rate of fatality. Distant-organ metastasis and regional lymph node metastasis are the major cause of mortality from the oral SCC. Oral lichen planus is regarded as a potential condition for malignant transformation, and thus dysplasia or carcinoma could arise from oral lichen planus (WHO, 1997).

Matrix metalloproteinases (MMPs) are a highly regulated superfamily of enzymes that degrade almost all extracellular matrix and basement membrane components, processes which are essential for invasion and subsequent metastasis (Liotta et al, 1980; Tryggvason et al, 1987; Chambers and Matrisian, 1997). Among the MMPs, type I collagenase (MMP-1 or interstitial collagenase) degrades the fibrillar collagens and thus is important for the tumour traversing the extracellular space. A 72-kDa type IV collagenase (MMP-2) and a 92-kDa type IV collagenase (MMP-9) degrade type IV collagen present in basement membranes and have been implicated in the invasion of several tumour types, including skin and breast cancers (Fessler et al, 1984; Murphy et al, 1989; Monteagudo et al, 1990; Pyke et al, 1992). MMP-2 and MMP-9 also degrade native type V, VII and X collagens, fibronectin, elastin and gelatin (Seltzer et al, 1989; Senior et al, 1991).

The activity of MMPs is regulated, in part, by tissue inhibitors of metalloproteinases (TIMPs) (Docherty and Murphy, 1990; Stetler-Stevenson et al, 1990). Imbalances in the extracellular activities of MMPs and TIMPs have been linked with pathological tissue destruction seen in cancer, arthritis and cardiovascular disease (Liotta et al, 1991; Brinckerhoff 1992; Armstrong et al, 1994). TIMPs have potent antiangiogenic properties and can suppress tumour invasion and metastasis (Liotta et al, 1991; DeClerck et al, 1992). Among the TIMPs the best known are TIMP-1, -2 and -3 (Welgus and Stricklin, 1983; Stetler-Stevenson et al, 1989; Apte et al, 1994). More recently, an additional member of the TIMP family, called TIMP-4, has been isolated (Greene et al, 1996).

Regarding the in vivo involvement of MMPs and their inhibitors in human oral cancers, little information has been

*Received 14 July 1997*
*Revised 10 November 1997*
*Accepted 12 November 1997*

*Correspondence to*: T Salo, Institute of Dentistry, Department of Oral Maxillofacial Surgery, Aapistie 3, SF-90220 Oulu, Finland

*The first two authors contributed equally to this work

**Figure 1** mRNA expression of MMP-1, MMP-2, TIMP-1 and TIMP-3 in oral squamous cell carcinoma studied by in situ hybridization. MMP-1 mRNA is expressed by stromal cells surrounding the neoplastic islands (arrows) (**A** and **B**). MMP-2 gene expression is detected in fibroblast-like cells of tumoral stroma (**C** and **D**). TIMP-1 mRNA is found in some stromal cells (**E** and **F**), whereas a clear TIMP-3 mRNA expression is seen in stromal cells surrounding the neoplastic islands (**G** and **H**). (Magnifications × 250; **B**, **D**, **F** and **H** are dark-field images)

*© Cancer Research Campaign 1998*

presented. In SCCs, MMP-1 mRNA expression has been shown in stromal cells and in cancer cells of tumour islands (Polette et al, 1991; Gray et al, 1992; Muller et al, 1993). In human skin cancers, MMP-9 is synthesized by the tumour cells, whereas MMP-2 is derived from the stromal cells surrounding the neoplastic islands (Pyke et al, 1992). Among the TIMPs, in oral SCC, a low TIMP-1 gene expression has been reported in fibrous connective tissue (Gray et al, 1992) and also in well-differentiated cancer cells and in endothelial cells, whereas TIMP-2 mRNA was localized in a few stromal cells near invasive cancer cells and in endothelial cells as well (Polette et al, 1993). To our knowledge there are no earlier reports from TIMP-3 expression in oral lesions.

MMPs are thought to be key enzymes mediating cancer invasion, but their roles in premalignant lesions and conditions are less clear. With this background we have now investigated the mRNA expression and protein distribution of MMP-1 and -2, and TIMP-1, -2 and -3 in oral SCC and lymph node metastasis. We further compared the MMP and TIMP expression in carcinoma with their expression in oral epithelial dysplasias, lichen planus and normal buccal mucosa. The aim of the zymography and enhanced chemiluminescence (ECL) Western blots was to find out if there were some differences in MMP amounts and the degree of conversion of latent MMPs to active counterparts between SCC, lichen planus and normal oral mucosa.

### MATERIALS AND METHODS

Oral biopsy specimens of ten SCCs, ten lymph node metastases, ten lichen planus, six epithelial dysplasias and four normal buccal mucosa were included in the study. SCCs were graded histologically as well–moderately ($n = 6$) and poorly differentiated ($n = 4$) (Wahi et al, 1971). Dysplasias were graded as mild ($n = 4$) and moderate ($n = 2$) (Who Collaborating Centre for Oral Precancerous Lesions, 1978). Diagnosis of lichen planus was based on clinical and histopathological criteria. The investigation was approved by the Ethics Committee of the University of Oulu.

### RNA probes and in situ hybridization

Paraffin sections (4 µm) were used for in situ hybridization. A detailed description of the preparation of paraffin sections for in situ hybridization has been described previously (Autio-Harmainen et al, 1992). A 1017-bp *Eco*RI–*Bam*HI fragment of MMP-1 cDNA clone (Goldberg et al, 1986) was ligated in the pGEM 4Z vector. A 635-bp *Sca*I–*Sac*I fragment of human MMP-2 cDNA clone (Huhtala et al, 1990) was cloned into the M13 polylinker site of pSP64 and pSP65 vectors. A 626-bp *Bam*HI–*Hin*dIII fragment of TIMP-1 coding sequence was cut from the pUC 19 vector. Originally, the TIMP-1 cDNA, described by Docherty et al (1985) and Carmichael et al (1986) was amplified by polymerase chain reaction (PCR) using the available sequence data from the GenBank. This cDNA fragment was ligated in pGEM 4Z vector. A 388-bp *Eco*RI–*Kpn*I fragment of the human TIMP-2 cDNA clone, described by Stetler-Stevenson et al (1990) from pSS 38 vector was ligated in pGEM 4Z vector. A 500-bp *Eco*RI–*Pst*I fragment of the human TIMP-3 cDNA clone described by Apte et al (1994) was ligated in pGEM 4Z vector. Vectors were linearized with suitable restriction enzymes, and for transcription a riboprobe trancription kit (Promega) was used and the transcripts were labelled by [$^{35}$S]UTP to specific activities of $3 \times 10^6$ c.p.m. per 40 µl. Solutions were treated with 0.1% diethylpyrocarbonate. All the probes have been tested by Northern hybridization (data not shown) and by sequencing the whole cDNA.

In situ hybridization with antisense and sense RNA probes has been described previously (Autio-Harmainen et al, 1992). The hybridizations were performed at 50°C, followed by washing, autoradiography for 7–15 days, and staining of the sections with haematoxylin and eosin.

### Immunohistochemical staining

Paraffin sections (6 µm) were washed in phosphate-buffered saline (PBS) for 10 min. Non-specific binding of IgGs was blocked using normal rabbit serum (1:20 in PBS with 0.1% bovine serum albumin, BSA). The sections were incubated overnight with primary antibody at 4°C. Negative controls were stained with PBS and normal mouse or rabbit IgG instead of the primary antibody. The sections were then incubated (30 min) with biotinylated anti-mouse or anti-rabbit IgG secondary antibody (Vector Elite kit, Abbott, Chicago, IL, USA), the avidin–peroxidase complex (30 min), and the substrate (0.05% 3,3-diaminobenzidine tetra hydrochloride (DAB) in Tris buffer, pH 7.4); (Sigma, Poole, UK) (4 min). A DAB enhancing solution (Vector Laboratories, Burlingame, CA, USA) was used according to product protocol to intensify stain in some stainings. For morphological observation haematoxylin routine histological stain was used.

Primary antibodies against the following molecules were used: polyclonal antibodies against MMP-1, 1:100 (Goldberg et al, 1986); MMP-2, 1:200 (Wallon and Overall, 1997); TIMP-1, 1:200 (Bodden et al, 1994); TIMP-3, 1:500 (Triple Point Biologics, OR, USA) and monoclonal antibody against TIMP-2, 1:200 (Höyhtyä et al, 1994).

### Assay of gelatinase by zymography

Gelatinases from 25-µm-thick and same size frozen tissue sections homogenized in sample buffer were assayed using the zymography method described previously (Salo et al, 1991). Zymography was performed in 1.5-mm 10% polyacrylamide slab gels containing 1 mg ml$^{-1}$ gelatin labelled with 2-methoxy-2,4-diphenyl-3(2H)-furanone (Fluka, Ronkonkoma, NY, USA). The gels were photographed under long-wave ultraviolet illumination. We estimated cleavage rates by densitometric scanning, from negatives of the photographed gels, using a Computing Densitometer model 300A (Molecular Dynamics, CA, USA).

### Western blotting

For Western analysis, 25-µm-thick and same size frozen tissue sections homogenized to sample buffer were run on 10% SDS-polyacrylamide gel, and transferred to nitrocellulose filters (Schleicher & Schuell, Dassel, Germany) or Immobilon-P PVDF transfer membrane (Millipore Corporation, Bedford, MA, USA). Non-specific binding was blocked by incubation with tris-buffered saline (TBS) supplemented with 10% non-fat dry milk (Difco Laboratories, Detroit, MI, USA) for 60 min. After washing, the filters were incubated with primary antibodies (MMP-1, MMP-2, TIMP-1, TIMP-2, TIMP-3) (1:500) overnight at 4°C. After washing, the filters were incubated with biotinylated secondary antibody (1:500, Dako, Glostrup, Denmark) for 1 h at room temperature. After washing, the filters were incubated with the

© Cancer Research Campaign 1998

**Figure 2** mRNA expression of MMP-1 in oral epithelial dysplasia studied by in situ hybridization. MMP-1 mRNA is detected in few fibroblasts under the basement membrane (arrows) (**A** and **B**). (Magnifications × 250; **B** is dark-field image)

avidin–peroxidase complex for 45 min. ECL Chemiluminescence Western Blotting detection kit (Amersham LifeScience, Buckinghamshire, UK) was used as described in the product protocol. In the method, the chemiluminescence reaction produced by ECL reagents was detected using autoradiography.

### RESULTS

### In situ hybridization

In situ hybridization experiments were carried out on ten oral SCCs, ten lymph node metastases, six epithelial dysplasias, ten lichen planus and four normal buccal mucosa, using antisense RNA probes specific to MMP-1, -2, and TIMP-1, -2 and -3.

In all oral SCC samples, MMP-1 mRNA was observed in fibroblastic cells of tumoral stroma (Figure 1A and B). In two out of ten cases, the expression was also detected in cancer cells in which MMP-1 gene expression was restricted to the peripheral cells of neoplastic islands (data not shown). In lymph node metastasis, the expression was detected in fibroblast-like cells in tumoral stroma (data not shown). In oral lichen planus and dysplastic lesions, the MMP-1 mRNA expression was low. In two out of six epithelial dysplasias and in one out of ten lichen planus studied, few fibroblasts under the basement membrane were positive (Figure 2A and B). In normal oral mucosa, the expression for MMP-1 was negative.

MMP-2 mRNA expression was noted in fibroblasts surrounding the tumour cells in invasive SCC (Figure 1C and D) and lymph node metastasis. Malignant cells were negative in all cases. In oral normal mucosa and lichen planus, the mRNA expression was not detected (data not shown). In two out of six dysplastic lesions studied, few positive fibroblasts were seen (data not shown).

In SCC and in lymph node metastasis, modest TIMP-1 expression was noted in stromal cells surrounding the neoplastic islands (Figure 1E and F). No TIMP-1 mRNA expression was detected in oral normal mucosa, lichen planus or dysplastic lesions (data not shown). TIMP-2 gene expression was not detected in any of the samples studied (data not shown).

In invasive SCC and lymph node metastasis, a clear TIMP-3 expression was noted in stromal cells surrounding the neoplastic islands (Figure 1G and H). Little or no mRNA for TIMP-3 was detected in oral normal mucosa, lichen planus or dysplastic lesions (data not shown).

As a negative control, sense RNA probes were applied to adjacent sections of all specimens. In these sections, no signal was seen (data not shown).

### Immunohistochemistry

Oral SCCs and lymph node metastasis were studied by immunohistochemical staining using specific antibodies to MMPs (MMP-1 and -2) and TIMPs (TIMP-1, -2 and -3).

In SCC and lymph node metastasis, there was a detectable staining reaction for MMP-1 in stromal cells and also in some carcinoma cells (Figure 3A). Immunostaining for MMP-2 was detected in the peripheral cell layer in neoplastic islands and in some fibroblast-like cells of tumoral stroma (Figure 3B). Endothelial cells stained consistently well with MMP-2 antibody. In lymph node metastasis, positive MMP-2 immunostaining was seen in some tumour cells (Figure 3C). A weak positive staining reaction for TIMP-1 was located in tumoral stroma, whereas the staining for TIMP-2 was negative (data not shown). There was a clear immunostaining reaction for TIMP-3 in stromal cells surrounding the neoplastic islands (Figure 3D).

### Zymography

Zymography of five oral SCC samples is shown in Figure 4A. Strong pro-MMP-9 was found in all SCC samples studied, whereas active MMP-9 species were weaker and seem to vary between different samples. In the studied carcinomas, MMP-9 dominated MMP-2, and active MMP-2 species dominated latent species. Clearly, more gelatinases were detected in SCC in relation to lichen planus and normal mucosa (Figure 4B). In normal mucosa, gelatinases existed predominantly as inactive latent proforms. In lichen planus, gelatinase intensities were between normal mucosa and carcinoma sample.

In ECL-Western blotting with anti-MMP-2, both latent and active species were identified and their intensities corresponded to those detected by zymography. With anti-MMP-1, the strongest band was seen in oral SCC compared with lichen planus and normal mucosa. In ECL-Western blots with TIMP-antibodies, small 20–30 kDa

 © Cancer Research Campaign 1998

**Figure 3** Immunohistochemical stainings of MMP-1, MMP-2, and TIMP-3 in oral squamous cell carcinoma. A detectable staining reaction for MMP-1 is seen in stromal cells and also in some carcinoma cells (**A**). Immunostaining for MMP-2 was detected in peripheral cell layer in neoplastic islands and in some fibroblast-like cells of tumoral stroma (**B**). In lymph node metastasis, positive MMP-2 immunostaining was seen in some tumour cells (**C**). There was a clear immunostaining reaction for TIMP-3 in stromal cells surrounding the neoplastic islands (**D**). (In D a DAB enhancing solution was used; magnifications × 250)

molecular weight bands were not detected. SDS–PAGE analysis using Coomassie brilliant blue staining demonstrated similar protein content of the sample groups studied (data not shown).

### DISCUSSION

We studied, using in situ hybridization and immunohistochemistry, the expression and distribution of MMP-1 and -2, and their tissue inhibitors (TIMP-1, -2 and -3) in oral SCCs and lymph node metastasis. The expression was also studied in oral lichen planus, epithelial dysplasias and normal buccal mucosa. Tissue samples were also investigated using zymography and ECL-Western blots to find out whether there are any differences in MMP amounts and the degree of conversion of latent MMPs to active counterparts between SCC, lichen planus and normal oral mucosa.

To our knowledge there are no earlier reports from TIMP-3 distribution in oral lesions. In oral SCC and lymph node metastasis, strong TIMP-3 expression was found in stromal cells surrounding the neoplastic islands. In addition to inhibitory activity, it has been suggested that TIMP-3 has a possible role in connective tissue turnover and remodelling (Uría et al, 1994). However, Sun et al (1996) have recently shown that TIMP-3 overexpression in mouse epidermal cells had no effect on growth, tumorigenity or invasion and did not reverse tumour cell phenotype in tumour cells lacking endogenous TIMP-3.

Previous reports have indicated that MMP-1 is synthesized by stromal cells and in some cases also by tumour cells in oral SCC (Gray et al, 1992; Muller et al, 1993). In our studies from oral SCC and lymph node metastasis, stromal cells and peripheral cells in neoplastic islands expressed MMP-1 mRNA. There was a detectable immunostaining reaction for MMP-1 in stromal cells and also in some carcinoma cells. MMP-1 is highly active against interstitial collagen and is thus important for the tumours to traverse the extracellular space and tumour invasion.

Our results of MMP-2 mRNA expression in oral SCC, lymph node metastasis, lichen planus and epithelial dysplasias are in line

© Cancer Research Campaign 1998

**Figure 4** Zymography of oral squamous cell carcinoma, lichen planus and normal mucosa. (**A**) Strong pro-MMP-9 is found in all squamous cell carcinoma samples studied, whereas active MMP-9 species are weaker and seem to vary between different samples. In these carcinomas, MMP-9 dominates MMP-2, and active MMP-2 species dominate latent species. (**B**) Clearly more gelatinases are detected in squamous cell carcinoma (lane 1) than in lichen planus (lane 2) and normal oral mucosa (lane 3). In normal mucosa, gelatinases exist predominantly as inactive latent proforms. In lichen planus, gelatinase intensities were between normal mucosa and carcinoma sample with Pyke et al (1992), who showed by using in situ hybridization that a 72-kDa type IV collagenase is derived from the stromal cells surrounding the tumour cells in human skin cancers. The mRNA expression of MMP-2 in lymph node metastasis correlates with the expression in oral SCC, and thus confirms and further extends the role of this enzyme in tumour invasion. However, in our study clear immunostaining for MMP-2 was detected in peripheral cell layer in neoplastic islands and in some fibroblast-like cells of tumoral stroma in SCC. In epithelial dysplasias and lichen planus, slight MMP-1 and -2 mRNA expression was locally found under the basement membrane. These enzymes are capable of degrading the basement membrane components and this could thus, at least in theory, help in cancer progression.

According to previous studies of oral SCC, hyperplastic and dysplastic lesions, TIMP-1 gene expression was low and negligible with grains scattered throughout the fibrous connective tissue of all non-neoplastic epithelia examined, as it was in all the neoplastic tissues investigated (Gray et al, 1992). However, in epidermoid head and neck carcinomas, Polette et al (1993) found TIMP-1 mRNA expression in well-differentiated cancer cells and in endothelial cells, whereas TIMP-2 mRNA was localized in a few stromal cells near invasive cancer cells and in endothelial cells as well. In our present investigation, in SCC and lymph node metastasis, TIMP-1 mRNA expression was detected in some stromal cells surrounding the neoplastic islands and weak positive immunostaining was located in tumoral stroma. TIMP-2 was negative in all carcinoma and lymph node metastasis studied. MMP and TIMP expressions were consistently low in lichen planus, and mild and moderate epithelial dysplasias studied. In normal buccal mucosa, the expressions were negative.

We also homogenized frozen tissue sections directly into sample buffer without any purification. When gelatinolytic activities present in oral SCC, lichen planus and normal mucosa were compared, significantly more gelatinase activities were detected in carcinoma than in lichen planus and normal mucosa. In carcinoma, the active MMP-2 species clearly dominated the latent ones, but in normal mucosa, intensities were vice versa. In carcinoma, latent MMP-9 species dominated active ones, whereas in normal mucosa the overall amount of MMP-9 was weak. In lichen planus, gelatinase intensities were between normal mucosa and carcinoma sample. In zymography of SCC, many gelatinases with different molecular sizes were found, and the ECL-Western blot was used to identify MMP-2. Latent and active MMP-2 were identified and their intensities corresponded to zymographic findings. With anti-MMP-1, the strongest band was seen in oral SCC compared with lichen planus and normal mucosa. With TIMP-antibodies, small 20–30 kDa molecular weight bands were not found in homogenized frozen tissue samples. In all ECL-Western blots carried out, many high-molecular-weight bands were found. These bands did not correspond to the well-known molecular weights of MMPs and TIMPs studied. The bands might be the results of the protein–protein interactions (i.e. MMP–TIMP complexes) in tissue samples and there might also be some non-specific binding of ECL reagents.

MMPs have long been associated with metastasis, and there is no doubt that they are major functional contributors to the metastatic process. It has been suggested that MMPs are important in creating and maintaining an environment that supports the initiation and maintenance of growth of primary and metastatic tumours. MMPs and their inhibitors appear to be important regulators of the growth of tumours, both at the primary state and as metastases (Chambers and Matrisian, 1997). Some of these activities may be derived from tumour-associated host tissues as a host response to the presence of invasive malignant cells, as suggested by Poulsom et al (1993).

It seems that the MMPs and TIMPs studied are involved in extracellular matrix remodelling during cancer invasion in oral SCC. We found slight but clear MMP-1 and MMP-2 mRNA expression in oral premalignancies; these expressions were weaker in relation to oral SCC. We conclude that in oral SCC similarly to human skin and other carcinoma types, regulation of extracellular matrix remodelling during cancer invasion is the result of a concerted action of MMPs and TIMPs.

### ACKNOWLEDGEMENTS

This study was partially supported by grants from the Medical Research Council of the Academy of Finland, the Finnish Dental Society, the Cancer Foundation of Northern Finland, the Finnish Cancer Foundation, Research and Science Foundation of Farmos and EVO grants from Helsinki and Oulu University Hospitals.

### REFERENCES

Apte SS, Mattei M-G and Olsen BR (1994) Cloning of the cDNA encoding human tissue inhibitor of metalloproteinases-3 (TIMP-3) and mapping of the TIMP3 gene to chromosome 22. *Genomics* **19**: 86–90

Armstrong PW, Moe GW, Howard RJ, Grima EA and Cruz TF (1994) Structural remodelling in heart failure: Gelatinase induction. *Can J Cardiol* **10**: 214–220

Autio-Harmainen H, Hurskainen T, Niskasaari K, Höyhtyä M and Tryggvason K (1992) Simultaneous expression of 70 kilodalton type IV collagenase and type IV collagen α1(IV) chain genes by cells of early human placenta and gestational endometrium. *Lab Invest* **67**: 191–200

Bodden MK, Harber GJ, Birkedal-Hansen B, Windsor LJ, Caterina NCM, Engler JA and Birkedal-Hansen H (1994) Functional domains of human TIMP-1 (tissue inhibitor of metalloproteinases). *J Biol Chem* **269**: 18943–18952

© Cancer Research Campaign 1998

Brinckerhoff CE (1992) Regulation of metalloproteinase gene expression: implications for osteoarthritis. *Crit Rev Eukaryot Gene Expr* **2**: 145–164

Carmichael DF, Sommer A, Thompson RC, Anderson DC, Smith CG, Welgus HG and Stricklin GP (1986) Primary structure and cDNA cloning of human fibroblast collagenase inhibitor. *Proc Natl Acad Sci USA* **83**: 2407–2411

Chambers AF and Matrisian LM (1997) Changing views of the role of matrix metalloproteinases in metastasis. *J Natl Cancer Inst* **89**: 1260–1270

DeClerck YA, Perez N, Shimada H, Boone TC, Langley KE and Taylor SM (1992) Inhibition of invasion and metastasis in cells transfected with an inhibitor of metalloproteinases. *Cancer Res* **52**: 701–708

Docherty AJP and Murphy G (1990) The tissue metalloproteinase family and the inhibitor TIMP: a study using cDNAs and recombinant proteins. *Ann Rheum Dis* **49**: 469–479

Docherty AJP, Lyons A, Smith BJ, Wright EM, Stephens PE and Harris TJR (1985) Sequence of human tissue inhibitor of metalloproteinases and its identity to erythroid-potentiating activity. *Nature* **318**: 66–69

Fessler LI, Duncan KG, Fessler JH, Salo T and Tryggvason K (1984) Characterization of the procollagen IV cleavage products produced by a specific tumor collagenase. *J Biol Chem* **259**: 9783–9789

Goldberg GI, Wilhelm SM, Kronberger A, Bauer EA, Grant GA and Eisen AZ (1986) Human fibroblast collagenase. Complete primary structure and homology to an oncogene transformation-induced rat protein. *J Biol Chem* **261**: 6600–6605

Gray ST, Wilkins RJ and Yun K (1992) Interstitial collagenase gene expression in oral squamous cell carcinoma. *Am J Pathol* **141**: 301–306

Greene J, Wang M, Liu YE, Raymond LA, Rosen C and Shi YE (1996) Molecular cloning and characterization of human tissue inhibitor of metalloproteinase 4. *J Biol Chem* **271**: 30375–30380

Huhtala P, Chow LT and Tryggvason K (1990) Structure of the human type IV collagenase gene. *J Biol Chem* **265**: 11077–11082

Höyhtyä M, Fridman R, Komarek D, Porter-Jordan K, Stetler-Stevenson WG, Liotta LA and Liang C-M (1994) Immunohistochemical localization of matrix metalloproteinase 2 and its specific inhibitor TIMP-2 in neoplastic tissues with monoclonal antibodies. *Int J Cancer* **56**: 500–505

Liotta LA, Tryggvason K, Garbisa S, Hart I, Foltz CM and Shafie S (1980) Metastatic potential correlates with enzymatic degradation of basement membrane collagen. *Nature* **284**: 67–68

Liotta LA, Steeg PS and Stetler-Stevenson WG (1991) Cancer metastasis and angiogenesis: an imbalance of positive and negative regulation. *Cell* **64**: 327–336

Monteagudo C, Merino MJ, San-Juan J, Liotta LA and Stetler-Stevenson WG (1990) Immunohistochemical distribution of type IV collagenase in normal, benign, and malignant breast tissue. *Am J Pathol* **136**: 585–592

Muller D, Wolf C, Abecassis J, Millon R, Engelmann A, Bronner G, Rouyer N, Rio M-C, Eber M, Methlin G, Chambon P and Basset P (1993) Increased stromelysin 3 gene expression is associated with increased local invasiveness in head and neck squamous cell carcinomas. *Cancer Res* **53**: 165–169

Murphy G, Ward R, Hembry RM, Reynolds JJ, Kühn K and Tryggvason K (1989) Characterization of gelatinase from pig polymorphonuclear leucocytes. A metalloproteinase resembling tumour type IV collagenase. *Biochem J* **258**: 463–472

Polette M, Clavel C, Muller D, Abecassis J, Binninger I and Birembaut P (1991) Detection of mRNAs encoding collagenase I and stromelysin 2 in carcinomas of the head and neck by *in situ* hybridization. *Invas Metastas* **11**: 76–83

Polette M, Clavel C, Birembaut P and DeClerck YA (1993) Localization by *in situ* hybridization of mRNAs encoding stromelysin 3 and tissue inhibitors of metallo-proteinases TIMP-1 and TIMP-2 in human head and neck carcinomas. *Path Res Pract* **189**: 1052–1057

Poulsom R, Hanby AM, Pignatelli M, Jeffery RE, Longcroft JM, Rogers L and Stamp GWH (1993) Expression of gelatinase A and TIMP-2 mRNAs in desmoplastic fibroblasts in both mammary carcinomas and basal cell carcinoma of the skin. *J Clin Pathol* **46**: 429–436

Pyke C, Ralfkiaer E, Huhtala P, Hurskainen T, Dano K and Tryggvason K (1992) Localization of messenger RNA for $M_r$ 72,000 and 92,000 type IV collagenases in human skin cancers by *in situ* hybridization. *Cancer Res* **52**: 1336–1341

Salo T, Lyons JG, Rahemtulla F, Birkedal-Hansen H and Larjava H (1991) Transforming growth factor-β1 up-regulates type IV collagenase expression in cultured human keratinocytes. *J Biol Chem* **266**: 11436–11441

Seltzer JL, Weingarten H, Akers KT, Eschbach ML, Grant GA and Eisen AZ (1989) Cleavage specificity of type IV collagenase (gelatinase) from human skin. Use of synthetic peptides as model substrates. *J Biol Chem* **264**: 19583–19586

Senior RM, Griffin GL, Fliszar CJ, Shapiro SD, Goldberg GI and Welgus HG (1991) Human 92- and 72-kilodalton type IV collagenases are elastases. *J Biol Chem* **266**: 7870–7875

Stetler-Stevenson WG, Krutzcsh HC and Liotta LA (1989) Tissue-inhibitor of metalloproteinase (TIMP-2). A new member of the metalloproteinase inhibitor family. *J Biol Chem* **264**: 17374–17378

Stetler-Stevenson WG, Brown PD, Onisto M, Levy AT and Liotta LA (1990) Tissue inhibitor of metalloproteinases-2 (TIMP-2) mRNA expression in tumor cell lines and human tumor tissues. *J Biol Chem* **265**: 13933–13938

Sun Y, Kim H, Parker M, Stetler-Stevenson WG and Colburn NH (1996) Lack of suppression of tumor cell phenotype by overexpression of TIMP-3 in mouse JB6 tumor cells: identification of a transfectant with increased tumorigenicity and invasiveness. *Anticancer Res* **16**: 1–7

Tryggvason K, Höyhtyä M and Salo T (1987) Proteolytic degradation of extracellular matrix in tumor invasion. *Biochim Biophys Acta* **907**: 191–217

Uría JA, Ferrando AA, Velasco G, Freije JM and López-Otín C (1994) Structure and expression in breast tumors of human TIMP-3, a new member of the metalloproteinase inhibitor family. *Cancer Res* **54**: 2091–2094

Wahi PN, Cohen B, Luthra UK and Torloni H (1971) *Histological typing of oral and oropharyngeal tumours. International histological classification of tumours. No 4.* World Health Organization: Geneva

Wallon UM and Overall CM (1997) The hemopexin-like domain (C domain) of human gelatinase A (matrix metalloproteinase-2) requires $Ca^{2+}$ for fibronectin and heparin binding. Binding properties of recombinant gelatinase A C domain to extracellular matrix and basement membrane components. *J Biol Chem* **272**: 7473–7481

Welgus HG and Stricklin GP (1983) Human skin fibroblast collagenase inhibitor. Comparative studies in human connective tissues, serum, and amniotic fluid. *J Biol Chem* **258**: 12259–12264

WHO Collaborating Centre for Oral Precancerous Lesions (1978) Definition of leukoplakia and related lesions: An aid to studies on oral precancer. *Oral Surg Oral Med Oral Pathol* **46**: 518–539

World Health Organization (1997) Histological typing of cancer and precancer of the oral mucosa. In *International Histological Classification of Tumours.* Second edition, World Health Organization: Geneva

© Cancer Research Campaign 1998